# Sex Differences in the Nicotinic Acetylcholine Receptor System of Rodents: Impacts on Nicotine and Alcohol Reward Behaviors

**DOI:** 10.3389/fnins.2021.745783

**Published:** 2021-09-21

**Authors:** Janna K. Moen, Anna M. Lee

**Affiliations:** ^1^Graduate Program in Neuroscience, University of Minnesota Twin Cities, Minneapolis, MN, United States; ^2^Department of Pharmacology, University of Minnesota Twin Cities, Minneapolis, MN, United States

**Keywords:** nicotinic acetylcholine receptor, sex differences, alcohol, nicotine, addiction

## Abstract

Alcohol and nicotine are the two most widely used and misused drugs around the world, and co-consumption of both substances is highly prevalent. Multiple lines of evidence show a profound effect of sex in many aspects of alcohol and nicotine reward, with women having more difficulty quitting smoking and showing a faster progression toward developing alcohol use disorder compared with men. Both alcohol and nicotine require neuronal nicotinic acetylcholine receptors (nAChRs) to elicit rewarding effects within the mesolimbic system, representing a shared molecular pathway that likely contributes to the frequent comorbidity of alcohol and nicotine dependence. However, the majority of preclinical studies on the mechanisms of alcohol and nicotine reward behaviors utilize only male rodents, and thus our understanding of alcohol and nicotine neuropharmacology relies heavily on male data. As preclinical research informs the development and refinement of therapies to help patients reduce drug consumption, it is critical to understand the way biological sex and sex hormones influence the rewarding properties of alcohol and nicotine. In this review, we summarize what is known about sex differences in rodent models of alcohol and nicotine reward behaviors with a focus on neuronal nAChRs, highlighting exciting areas for future research. Additionally, we discuss the way circulating sex hormones may interact with neuronal nAChRs to influence reward-related behavior.

## Introduction

The study of both male and female animal subjects in biomedical research has increased in recent years, with results highlighting the complex interaction between biological sex, sex hormone signaling, and environmental stimuli. These sex differences can have multiple origins, including cellular differentiation and development as well as epigenetic modifications, which have been comprehensively reviewed elsewhere (McCarthy and Arnold, 2011; McCarthy and Nugent, 2013; McCarthy et al., 2015; Manoli and Tollkuhn, 2018; Gegenhuber and Tollkuhn, 2019). There are also well-established sex differences observed in neural reward circuitry, both at the organizational level and in regards to hormone signaling (Becker et al., 2012; Yoest et al., 2014; Meitzen et al., 2018). Work in the human laboratory and epidemiological studies have provided strong evidence of sex and gender differences in both alcohol and nicotine dependence. Women tend to have more difficulty quitting smoking and show faster progression toward developing an alcohol use disorder compared with men (Erol and Karpyak, 2015; Agabio et al., 2016; Verplaetse et al., 2018). While alcohol and nicotine are two of the most commonly abused substances worldwide, there is also high comorbidity between the two substance use disorders, with epidemiological studies suggesting that up to 92% of individuals with nicotine dependence also meet the criteria for alcohol use disorder (Van Skike et al., 2016). Neuronal nicotinic acetylcholine receptors (nAChRs) have been implicated in the addictive properties of both alcohol and nicotine, highlighting nAChRs as important physiological targets for both substances (Verplaetse and McKee, 2017). Preclinical rodent models have supported nAChRs as a common substrate for alcohol and nicotine reward and a potential therapeutic target to treat comorbid alcohol and nicotine use disorders (Hendrickson et al., 2013). However, at the preclinical level female animal subjects are historically underrepresented, with a landmark meta-analysis from 2011 finding neuroscience publications in particular included both sexes at a rate of only ∼20% (Beery and Zucker, 2011). More recent analyses have shown moderate improvements with ∼50% of neuroscience publications reporting the use of both male and female subjects in 2017, although only 15% of these studies using both sexes explicitly included sex as an experimental variable, and 16% of all neuroscience studies analyzed failed to report the sex of animal subjects altogether (Mamlouk et al., 2020; Woitowich et al., 2020). Similarly, relatively few studies of the nAChR system explicitly discuss sex as a biological variable: a PubMed search with the keyword “nicotinic receptor” lists over 22,400 publications from 1990 to 2021, while using the keywords “nicotinic receptor” and “sex” returns only ∼320 results. Presented here is an overview of the current knowledge of sex differences in the nAChR system in adult rodents, the resulting influences on alcohol and nicotine reward behaviors, and areas in which future studies can focus to provide critical information on the role of sex in alcohol and nicotine reward.

## The Nicotinic Acetylcholine Receptor System at a Glance

Neuronal nAChRs are widely expressed pentameric cation channels, consisting of 5 subunits that assemble around a central ion pore. Upon agonist binding, nAChRs permeate cations such as Na^+^, K^+^, and Ca^2+^ into the cell, depolarizing the membrane potential of the cell. As neuronal nAChRs are localized primarily on cell bodies and presynaptic terminals, they are poised to directly modulate cell excitability and neurotransmitter release. The nAChR family is also extremely diverse, with 11 different subunits (α2–7, 9–10; β2–4) expressed in the mammalian brain. The nAChR subunits have differing expression patterns in the brain, with some subunits widely expressed throughout the brain (such as α4 and β2), and other subunits primarily expressed in a few brain regions (such as α6 and β3). Importantly, the subunit composition of an assembled nAChR confers distinct pharmacological and biophysical properties (for a comprehensive review of nAChR structure and function see Albuquerque et al., 2009), and differences in subunit and subtype expression may suggest specific roles for nAChR subtypes in distinct brain regions. Various nAChR subtypes are found throughout many major systems in the brain including the mesolimbic dopamine system, where they contribute to affective behaviors including reward and addiction (Table 1).

**TABLE 1:** Summary of neuronal nAChR subunit expression in reward circuitry of adult rodents.

**Subunit**	**Expression profile**	**Notable properties**	**References**
α2	IPN		Gotti et al., 2009
α3	Basolateral amygdala, HPC, hypothalamus, MHb, thalamus, VTA		Wada et al., 1989; Champtiaux et al., 2002
α4	Widespread	High affinity, upregulated by chronic nicotine	Wada et al., 1989; Flores et al., 1992
α5	Caudate putamen, MHb, SNc, VTA	Does not participate in ACh binding	Wada et al., 1990; Exley et al., 2012
α6	Locus coeruleus, SNc, VTA	Downregulated by chronic nicotine	Le Novere, 1996; Lai et al., 2005
α7	Amygdala, HPC, hypothalamus, VTA	High Ca^2+^ permeability, rapid desensitization, forms homopentamers	Seguela et al., 1993
β2	Widespread	High affinity, upregulated by chronic nicotine	Wada et al., 1989; Flores et al., 1992
β3	LHb, MHb, SNc, VTA	Does not participate in ACh binding	Deneris et al., 1989
β4	IPN, MHb		Quick et al., 1999; Millar and Gotti, 2009

*HPC, hippocampus; IPN, interpeduncular nucleus; LHb, lateral habenula; MHb, medial habenula; SNc, substantia nigra pars compacta; VTA, ventral tegmental area.*

As their namesake suggests, nicotine is a potent and selective agonist at neuronal nAChRs. The ability of nicotine to elicit a rewarding response in animals is dependent upon nAChRs, as global nAChR blockade by the non-selective antagonist mecamylamine is sufficient to block nicotine-induced dopamine release (Nisell et al., 1994), locomotor sensitization (Biala and Staniak, 2010), and intracranial self-administration (Ikemoto et al., 2006). Different nAChR subtypes across the brain likely play different roles in modulating nicotine reward. Studies using global knockout animals have shown that the widely expressed high affinity α4β2^∗^ subtype (^∗^ indicating the possible presence of other subunits in the assembled heteropentamer) is important for nicotine consumption and reward (Picciotto et al., 1998; Pons et al., 2008; McGranahan et al., 2011; Liu et al., 2012; Sanjakdar et al., 2015; Bagdas et al., 2019). The α6 nAChR subunit, which is preferentially expressed in midbrain dopamine neurons and can be expressed with the α4 subunit in the α4α6^∗^ subtype, additionally plays a crucial role in regulating nicotine consumption and nicotine-induced dopamine release (Exley et al., 2008; Pons et al., 2008; Liu et al., 2012; Bagdas et al., 2019). Alternatively, nAChRs containing the α5 subunit have been implicated in the aversive properties of nicotine in rodents (Fowler et al., 2011). The mechanisms through which nAChR subtypes modulate nicotine reward and drug related behaviors are highly complex, with much remaining to be discovered (for recent review see Wills and Kenny, 2021). More recently, nAChRs have been implicated in the rewarding properties of alcohol. While the pharmacological targets of alcohol are numerous, a classic series of elegant experiments found that nAChRs specifically in the ventral tegmental area are required for alcohol-induced dopamine release in rodents (Blomqvist et al., 1993, 1997; Ericson et al., 1998, 2003). Further work demonstrated that alcohol potentiates currents through α4^∗^ nAChRs to influence alcohol reward (Liu et al., 2013). The smoking cessation drug varenicline can reduce alcohol consumption in rodents in a manner dependent upon α4^∗^ nAChRs, further implicating high-affinity nAChRs in alcohol’s pharmacological mechanism (Hendrickson et al., 2010). A more complete understanding of the interaction between alcohol, nicotine, and nAChRs will be crucial for the development of pharmacotherapies to treat comorbid alcohol and nicotine use disorders.

## Sex Differences in Nicotinic Acetylcholine Receptor Expression and Regulation

### Baseline Nicotinic Acetylcholine Receptor Expression

Nicotinic acetylcholine receptors are distributed throughout the mammalian brain yet relatively little is known about the mechanisms governing nAChR subtype transcription and translation *in vivo.* Accordingly, few studies to date have examined whether there are innate baseline sex differences in nAChR expression. One group used [^3^H]cytisine to measure high-affinity β2^∗^ nAChR binding sites following chronic nicotine administration in rats found that drug-naïve female rats had higher whole-brain nAChR density compared with male rats (Koylu et al., 1997). Male rats bred for high nicotine preference (nicotine-preferring rats) also display higher baseline α7 nAChR mRNA expression in the hippocampus and striatum compared with female nicotine-preferring rats, with no significant differences in α4, β2, or α5 transcripts between sexes (Gozen et al., 2016). Importantly, protein or surface receptor expression of nAChR subunits does not necessarily correspond to mRNA expression levels (Marks et al., 1992; Pauly et al., 1996) and studies of nAChR protein levels in native tissue are limited by a lack of subunit-specific antibodies (Moser et al., 2007). Further studies utilizing alternative methods such as autoradiography or fluorescent reporter animals will be useful to establish whether protein nAChR expression varies by sex in drug-naïve rodents.

In both rodents and humans, several nAChR subunits are found in closely linked genomic regions, referred to as gene clusters. Genes for the α6 and β3 nAChR subunits (*Chrna6* and *Chrnb3*, respectively) are located in a palindrome orientation on the same gene cluster, and prior work has shown they are likely co-regulated (Champtiaux et al., 2003; Lee and Messing, 2011). α6^∗^ nAChRs are predominately found on dopamine neurons in the ventral midbrain and directly regulate dopamine release and associated drug-related behaviors, but the mechanism through which these nAChRs are expressed at the cellular level is poorly understood. One candidate regulatory mechanism involves signaling by protein kinase C epsilon (PKCε), as our group has previously shown that genetic ablation of PKCε results in decreased α6 and β3 nAChR mRNA expression in male mice (Lee and Messing, 2011). However, further characterization of female mice revealed an oppositional effect of sex, with female PKCε knockout animals showing a threefold increase in α6 and β3 transcript expression in the ventral midbrain compared with WT littermates (Moen et al., 2021). Our data also showed a strong correlation between α6 and β3 transcript expression levels in the ventral midbrain across both WT and KO animals, further suggesting shared transcriptional mechanisms involving the *CHRNB3-CHRNA6* gene cluster. As α6^∗^ nAChRs in the ventral midbrain impact the addictive properties of both alcohol and nicotine, an effect of sex in their regulatory mechanism has important implications for drug development. Notably, we did not observe a sex difference in transcript expression levels of α6, β3, or PKCε itself in PKCε WT animals (Moen et al., 2021). It is possible that the regulation of α6 and β3 nAChR transcripts occurs through distinct PKCε-medicated mechanisms in male and female animals that arise following sexual differentiation of the brain. The mechanism through which PKCε regulates nAChR expression in both male and female animals remains to be determined and is the subject of ongoing investigation by our group.

The PKCε WT animals are maintained on a 129SvJae/J background with a single generation cross to C57BL/6J (Khasar et al., 1999; Moen et al., 2021). C57BL/6J mice have unique phenotypes compared with the 129SvJae/J line, including decreased sensitivity to several drug classes and decreased anxiety-like behaviors (Homanics et al., 1999). It is possible that the nAChR expression profile in C57BL/6J and 129SvJae/J mice differ, contributing to these phenotypes. Indeed, strain-specific differences in α4 nAChR expression across multiple mouse strains have been reported in the hippocampus that correlate with strain sensitivity to nicotine, although these studies did not report the sex of animals used (Gahring et al., 2004; Gahring and Rogers, 2008). These data highlight the importance of considering animal strain as biological variable in addition to sex, especially when comparing data collected from animals with different genetic backgrounds.

### Drug-Induced Nicotinic Acetylcholine Receptor Expression

Following chronic nicotine exposure, the high affinity α4β2^∗^ nAChR subtype is widely reported to undergo a process of upregulation resulting in increased surface expression without impacting nAChR mRNA (Marks et al., 1992; Pauly et al., 1996; Govind et al., 2012). This nicotine-induced nAChR upregulation is thought to compensate for nicotine-induced receptor desensitization and is a potential mechanism underlying nicotine tolerance (Fenster et al., 1999), and as such represents an important area of study for nAChR neuropharmacology. A report from Koylu et al. (1997) found that high affinity nAChR binding sites measured using [^3^H]cytisine increased in male rats exposed to chronic nicotine injections while female rats showed no changes, suggesting a potential sex difference in cellular responses to chronic nicotine. These data align with more recent work in humans using single-photon emission computed tomography measuring β2^∗^ nAChR availability: while male smokers had higher β2^∗^ availability in the striatum, cortex, and cerebellum compared with male non-smokers, female smokers did not differ in their β2^∗^ nAChR availability compared with female non-smokers in any region (Cosgrove et al., 2012). Similar sex differences were reported in nicotine-preferring rats in which hippocampal α4 and β2 mRNA levels were elevated in males compared with females following a 6-week nicotine 2-bottle choice assay (Gozen et al., 2016). These data are intriguing and while they appear to conflict with prior reports of nAChR upregulation occurring independent of mRNA levels, they could also reflect a difference in experimental paradigm. Chronic nicotine consumption over many weeks may induce different cellular adaptations than 7 days of non-contingent nicotine injections, and the changes in mRNA expression may not reflect surface receptor expression, which was not measured in Gozen et al. (2016). On the contrary, another study in rats found no effect of sex in radiolabeled epibatidine sites following prolonged intravenous (IV) self-administration from a sample containing the left hemi-brain (Donny et al., 2000). This could potentially be due to differences in nAChR upregulation between brain regions: in rats administered daily nicotine injections, females showed increased epibatidine labeling in the NAc core and shell, whereas males had higher nAChR density in the caudate (Lenoir et al., 2015). Interestingly, the dose used in this study (0.4 mg/kg nicotine) was only able to condition a place preference in females and not in males, and the results may reflect a more generalized effect of sex on nicotine pharmacology (Lenoir et al., 2015). In the IPN, chronic nicotine exposure results in increased α5 transcript levels only in females, while α7 transcript was increased only in males (Correa et al., 2019). Another recent report utilized mice with fluorescently tagged α4 and α6 nAChR subunits to measure receptor expression in the VTA following nicotine conditioned place preference, finding no sex differences in the degree of α4^∗^ and α4α6^∗^ nAChR upregulation (Akers et al., 2020).

While there is general agreement in the nAChR literature supporting the upregulation of α4^∗^ nAChRs by nicotine, the effect of nicotine on α6^∗^ expression remains unclear. Studies using autoradiography have shown little effect of chronic nicotine on the expression of putative α6^∗^ nAChRs in rats implanted with osmotic minipumps (Nguyen et al., 2003), while another study employing nicotine self-administration reported a marked increase in α6 binding measured by α-conotoxin (Parker et al., 2004). On the contrary, several studies have shown a decrease in α6 binding in rodents following continuous nicotine infusion (Mugnaini et al., 2006; Perry et al., 2007) as well as chronic exposure through nicotine-treated drinking water (Perez et al., 2008). The α6 subunit is localized almost exclusively in dopamine neurons in the ventral midbrain and DA terminals in the striatum (Le Novere, 1996; Powers et al., 2013), where it drives DA release in the nucleus accumbens. More recent studies using [^3^H]dopamine release and fast-scan cyclic voltammetry have shown that chronic nicotine reduces striatal dopamine transmission, supporting the hypothesis that α6^∗^ nAChR expression and/or function is reduced after chronic nicotine treatment (Lai et al., 2005; Exley et al., 2013; Marks et al., 2014). Interestingly, the Perez et al. (2008) study also found that chronic nicotine consumption had different effects on α6^∗^ nAChR expression depending upon the presence of an additional α4 subunit in the assembled heteropentamer complex, noting a decrease in α6α4β2^∗^ nAChRs but an increase in α6(non-α4)β2^∗^ nAChRs in the striatum. This study highlights nAChR subunit composition and stoichiometry as additional factors influencing nicotine-induced changes in receptor expression. Notably, these studies either relied on male animals or do not report the sex of animal subjects, and as such the effect of chronic nicotine exposure on α6^∗^ nAChR expression in female animals remains unknown. While the mechanisms underlying nicotine-induced changes in nAChR expression are still not fully understood, it seems likely that the process varies considerably by subunit, receptor stoichiometry, brain region, and/or cell type, which could be further influenced by sex (summarized in Table 2). Elucidation of the mechanisms underlying the regulation of nAChR expression, both at baseline and following chronic nicotine exposure, may provide better insight into potential mechanistic sex differences.

**TABLE 2:** Summary of studies on brain nAChR expression in male and female rodents.

**Method**	**Animal**	**Result**	**References**
**Baseline nAChR expression**			
Whole-brain [^3^H]cytisine binding	Sprague-Dawley rats	↑ density in female vs. male rat.	Koylu et al., 1997
qPCR	Nicotine-preferring rats	↑α7 transcript in HPC and Str of males vs. females. *n.d.* in α4, β2, α5 transcript.	Gozen et al., 2016
**Nicotine-induced nAChR expression**			
Whole-brain [^3^H]cytisine following 15 daily injections (0.6 mg/kg)	Sprague-Dawley rats	Males: ↑ binding following chronic nicotine. Females: *n.d.* following chronic nicotine.	Koylu et al., 1997
Half-brain [^3^H]epibatidine binding following IVSA	Sprague-Dawley rats	*n.d.*	Donny et al., 2000
[^3^H]epibatidine binding following CPP (0.4 mg/kg)	Sprague-Dawley rats	Males: ↑ density in caudate. Females: ↑ density in NAc core and shell.	Lenoir et al., 2015
qPCR following nicotine 2-bottle choice	Nicotine-preferring rats	↑α4 and β2 transcript in hippocampus of males vs. females.	Gozen et al., 2016
qPCR following chronic nicotine (minipump)	Wistar rats	Males: ↑α7 transcript in IPN. Females: ↑α5 transcript in IPN.	Correa et al., 2019
Fluorescence following CPP (0.5 mg/kg)	α4-mCherry X α6-GFP mice	No effect of sex in α4* or α6α4* upregulation.	Akers et al., 2020

CPP, conditioned place preference; HPC, hippocampus; IVSA, intravenous self-administration; Str, striatum; VMD, ventral midbrain; *n.d.*, no difference. * indicates additional subunits in the nAChR.

Notably, a handful of reports have recently shown that alcohol can also facilitate the upregulation of neuronal nAChRs in rodents. One group used human M10 and rat PC12 cells *in vitro* expressing different nAChR subtypes. Nicotine applied to the cultures alone dose- and time-dependently upregulated [^3^H]epibatidine binding, with the addition of alcohol further increasing nAChR binding (Dohrman and Reiter, 2003). In this study, alcohol alone did not significantly change nAChR binding. Another group used [^3^H]cytisine binding to measure high-affinity nAChR binding in adolescent C57BL/6 mice that consumed nicotine as a sole source of fluid, with one group receiving alcohol injections every other day to mimic cyclic patterns of consumption. Alcohol and nicotine exposure combined upregulated [^3^H]cytisine binding in the cerebral cortex and midbrain moreso than nicotine alone in both male and female adolescent mice (Ribeiro-Carvalho et al., 2008). Interestingly, in the cerebral cortex, alcohol treatment alone was sufficient to increase nAChR binding sites (Ribeiro-Carvalho et al., 2008). Finally, a recent report used knock-in mice expressing a YFP-tagged α4 nAChR subunit to measure expression levels of the α4 subunit. Male mice injected with a large acute dose of alcohol showed a robust increase in α4-YFP expression in the nucleus accumbens and amygdala, with no changes observed in the ventral tegmental area or prefrontal cortex (Tarren et al., 2017). Although more research is clearly needed to investigate the interactions between nicotine and alcohol on nAChR expression, these intriguing studies suggest that nicotine and alcohol may act to alter nAChR signaling at a cellular level, highlighting another possible mechanism underlying the high level of comorbidity between alcohol and nicotine use disorders. Further work that includes both male and female adult animals will be necessary to understand whether alcohol-induced changes in nAChR expression may be influenced by sex.

## Sex Differences in Nicotine Behaviors

### Pharmacology

Following nicotine administration, female C57BL/6 mice show faster liver elimination of nicotine compared with male animals, and were subsequently less sensitive to nicotine-induced suppression of Y-maze activity (Hatchell and Collins, 1980). In ICR mice, there were no notable sex differences in nicotine’s ability to produce hypothermia, hypolocomotion, or seizure phenotypes (Damaj, 2001). As these are well characterized effects of acute nicotine, the comparable effects between sexes in ICR mice would suggest that nicotine potency is similar in male and female animals. However, in behavioral assays, female ICR mice were less sensitive than males to the anxiolytic and antinociceptive effects of acute nicotine measured by tail flick, hot plate, and elevated plus maze assays (Damaj, 2001). The sex differences observed in only a subset of assays would argue against potential sex differences in nicotine pharmacokinetics, instead suggesting sex differences in nAChR subtypes that mediate these responses. As the Damaj (2001) study did not measure brain or plasma nicotine levels, concrete conclusions regarding sex differences in nicotine pharmacology in ICR mice remain difficult. However, in Sprague-Dawley rats, repeated IV infusions of nicotine resulted in 10x higher plasma concentration of nicotine in females compared with males (Harrod et al., 2007). A similar result was observed in C57BL/6J mice, in which females had elevated plasma levels of the nicotine metabolite cotinine following 7 days of repeated injections compared with males (Nguyen et al., 2020). It is important to note that nicotine pharmacology and subsequent dosage selection varies considerably by both species and strain, complicating the comparison of results between different animals (Matta et al., 2007). As such, differences in nicotine pharmacokinetics and metabolism observed in rodents may not generalize to humans. Still, a more complete understanding of the way sex influences nicotine pharmacology in rodent models will be critical to interpret subsequent behavioral results in both male and female animals.

### Oral Consumption

Chronic oral nicotine consumption using a two-bottle choice procedure is a technically simple method for measuring long-term voluntary drug consumption. C57BL/6J mice in particular will consume significant quantities of nicotine in these procedures without requiring training or addition of sweetener to the drinking solutions, whereas the DBA/2J strain consumes significantly less (Locklear et al., 2012; O’Rourke et al., 2016; Bagdas et al., 2019). Within the high-drinking C57BL/6J strain adult females will consume more nicotine adjusted for body weight compared with males, an effect observed in multiple laboratories including our own (Klein et al., 2004; Glatt et al., 2009; Locklear et al., 2012; O’Rourke et al., 2016; Bagdas et al., 2019; DeBaker et al., 2020a). Although it remains unclear why female mice consume more nicotine than males, the persistence of this trend across laboratories and consumption procedures suggests a fundamental sex difference in nicotine and nAChR neurobiology in this strain.

The oral nicotine two-bottle choice assay is also particularly amendable to studies using genetically modified mice which are typically backcrossed onto a C57BL/6J background, although much of the original published work in nAChR knockout animals was performed exclusively in males. Recently, Bagdas et al. (2019) published a comprehensive study of oral nicotine consumption in several nAChR subunit knockout animals that were powered to detect potential sex differences. In mice lacking either α6 or β2 nAChRs, nicotine consumption was reduced at higher concentrations without an observed effect of sex. However, mice lacking the α7 subunit displayed a bidirectional sex difference, with female α7 knockouts consuming less nicotine compared with wild-type controls while male α7 knockouts consumed more nicotine compared with wild-types. The mechanism for this oppositional effect is not known. An in-depth comparative analysis of α7 nAChR expression and function across reward-related brain regions in male and female animals will be a necessary next step toward understanding how these receptors are differentially modulating nicotine consumption by sex. While bidirectional sex differences in reward-related behaviors is uncommon, our work examining the influence of PKCε on nAChR expression revealed a similar effect on nicotine consumption in male and female PKCε knockout mice. Male PKCε knockouts have decreased expression of α6 and β3 transcript and showed decreased nicotine consumption during the first week of a 4-week nicotine 2-bottle choice assay compared with wild-type littermates (Lee and Messing, 2011), whereas female PKCε knockouts showed increased consumption during the first week of the same procedure (Moen et al., 2021).

### Self-Administration

Intravenous self-administration (IVSA) is a powerful way to study the motivation and incentive of rodents to pursue nicotine infusions. Much like oral nicotine consumption, female rats typically show higher rates of nicotine self-administration than males (Chaudhri et al., 2005; Grebenstein et al., 2013; Flores et al., 2016, 2019), with some notable exceptions (see Table 3). In one study, female Sprague-Dawley rats did not differ significantly from males in number of active responses or number of earned infusions, but did exhibit faster acquisition of self-administration during fixed-ratio schedules as well as higher breakpoints on a progressive ratio schedule (0.02–0.09 mg/kg/infusion, FR5; Donny et al., 2000). These data indicate that female rats have a higher motivation to obtain nicotine compared with males. In contrast, a more recent report found male Sprague-Dawley rats will self-administer more nicotine than female animals (0.02 mg/kg/infusion, FR1), while no sex difference was reported for Long–Evans rats (Leyrer-Jackson et al., 2020). However, subsequent meta-analysis of rat IVSA studies supports the finding that female rats across strains self-administer more nicotine compared with males (Flores et al., 2019). Female Wistar rats will self-administer more nicotine than either males or ovariectomized females, while supplementation of estradiol in the gonadectomized females restored nicotine self-administration to the same levels as intact females (0.015–0.06 mg/kg/infusion, FR1; Flores et al., 2016). Importantly, a role of circulating hormones may not implicate variability across the estrous cycle, as previous studies found no effect of estrous stage on rates of nicotine self-administration (Donny et al., 2000) or used freely cycling female animals with similar variance compared with males (Grebenstein et al., 2013; Flores et al., 2016). As such, sex differences in nicotine consumption and self-administration in adult rodents may instead reflect structural and organizational differences following sexual differentiation of the brain.

**TABLE 3:** Summary of sex differences in highlighted nicotine behavioral studies.

**Behavior**	**Animal**	**Result**	**References**
Chronic nicotine 2-bottle choice	C57BL/6J mice	↑ nicotine consumption in females	O’Rourke et al., 2016; Bagdas et al., 2019
	DBA/2J mice		
	α7 KO mice	↓ consumption in female KO. ↑ consumption in male KO.	Bagdas et al., 2019
	α6 KO, β2 KO mice	↓ consumption in KO mice, no effect of sex.	
IVSA	Rat, multiple strains	↑ nicotine SA in females vs. males (meta-analysis).	Flores et al., 2019
	Sprague-Dawley rat	*n.d.* in earned infusions, lever presses. ↑ SA acquisition on FR schedule in females. ↑ breakpoints in females in PR schedule.	Donny et al., 2000
	Sprague-Dawley rat	↑ SA in males vs. females	Leyrer-Jackson et al., 2020
	Long–Evans rat	*n.d.* in SA	
	Wistar rat	↓ SA in OVX females, restored to intact levels with E2.	Flores et al., 2016
	Adolescent Sprague-Dawley rat	Males: ↓ SA in adulthood. Females: *n.d.* in SA in adulthood	Levin et al., 2003, 2007
Precipitated withdrawal: somatic	Wistar rat	*n.d.* in somatic withdrawal signs	Correa et al., 2019
	Wistar rat	↑ somatic withdrawal signs in males.	Tan et al., 2019
Precipitated withdrawal: EPM	Wistar rat	↑ in females vs. males	Correa et al., 2019; Flores et al., 2020
	Wistar rat, OVX	↑ with E2 supplementation, ↓ with progesterone supplementation.	Flores et al., 2020
Spontaneous withdrawal: EPM	Wistar rat	↑ in females vs. males, *n.d.* following OVX.	Torres et al., 2014

*IVSA, intravenous self-administration; SA, self-administration; FR, fixed ratio; PR, progressive ratio; OVX, ovariectomy; EPM, elevated plus maze; E2, estradiol; *n.d.*, no difference.*

Self-administration paradigms have also been used to study the effect of nicotine in adolescents. Adolescent male rats will self-administer more nicotine than adults but will steadily decrease their self-administration as they approach adulthood, an effect often attributed to changes in brain nAChR expression that occur during brain reorganization prior to adulthood (Levin et al., 2007). On the contrary, adolescent females will self-administer more nicotine than adult females with heightened nicotine self-administration persisting into adulthood (Levin et al., 2003), a finding which suggests that adolescent females may be particularly vulnerable to negative effects of nicotine exposure. Adolescent female rats will also acquire nicotine self-administration more readily than adolescent males and show differences in progressive ratio responding during estrous (Lynch, 2009), in contrast to female adult rats that do not vary during the estrous cycle (Donny et al., 2000). Cholinergic signaling, and by extension the actions of nicotine, play critical and complex roles during brain development which can also vary considerably by sex. These highlighted studies are only a small fraction of work published on the intersection of sex and nicotine on the developing brain, and we point interested readers to recent comprehensive reviews on the subject (Cross et al., 2017; Thorpe et al., 2020).

### Withdrawal

In rodent models, nicotine withdrawal is often induced by administration of the non-selective nAChR antagonist mecamylamine following prolonged nicotine consumption or non-contingent nicotine exposure. Such procedures of precipitated nicotine withdrawal induce somatic withdrawal symptoms and negative affect. Studies of somatic withdrawal signs are mixed, with some reporting no sex differences (Correa et al., 2019) and others showing male rats with higher somatic withdrawal scores compared with females (Tan et al., 2019). However, female rodents consistently show heightened sensitivity to the negative affect aspect of nicotine withdrawal. Female rats receiving continuous nicotine show higher corticosterone levels following mecamylamine-precipitated withdrawal, consistent with a heightened activation of the hypothalamic-pituitary adrenal axis associated with stress (Gentile et al., 2011). In the elevated plus maze, mecamylamine-precipitated withdrawal was also significantly more anxiogenic in females versus males (Correa et al., 2019). Notably, female rats also showed heightened corticosterone levels and anxiety at baseline compared with males (Gentile et al., 2011; Correa et al., 2019), which may reflect a more generalized susceptibility to exhibiting stress and anxiety-like responses. Supporting this framework, the Correa et al. (2019) study additionally found that female rats had higher concentrations of acetylcholine in the IPN both at baseline and during nicotine withdrawal alongside higher expression levels of the α5 nAChR subunit. As activation of nAChRs in the habenulo-interpeduncular pathway can drive aversion to nicotine and other salient stimuli (Fowler et al., 2011), heightened cholinergic signaling in the IPN in females represents a potential mechanism to account for the observed behavioral sex differences. As women who smoke have more difficulty achieving and maintaining abstinence (Verplaetse et al., 2018), further study on the interaction between sex and negative affect following both mecamylamine-precipitated and spontaneous nicotine withdrawal may provide crucial insight into better treatment options for smoking cessation.

## Sex Differences in Nicotinic Acetylcholine Receptor-Mediated Alcohol Behaviors

Similar to nicotine, female rodents tend to consume more alcohol than males in chronic 2 bottle choice procedures (O’Rourke et al., 2016; Priddy et al., 2017), binge drinking-in-the-dark paradigms (Szumlinski et al., 2019), and operant self-administration (Nieto and Kosten, 2017) with no observed impact of estrous stage (Priddy et al., 2017). Alcohol reward and related behaviors involve cholinergic signaling through nAChRs, although the vast majority of the pioneering studies on cholinergic modulation of alcohol reward utilized only male animals. While sex differences in generalized alcohol-related behaviors have been investigated in rodents, few studies have specifically addressed the role of nAChRs in alcohol reward using both male and female animals (summarized in Table 4), and those that have powered their analyses to detect sex differences report few effects of sex.

**TABLE 4:** Summary of sex differences in highlighted alcohol behavioral studies.

**Method**	**Animal**	**Result**	**References**
**Alcohol**			
Chronic alcohol 2-bottle choice	C57BL/6J mice	↑ consumption in female vs. male.	O’Rourke et al., 2016
	Wistar and Long–Evans rats		Priddy et al., 2017
Intermittent alcohol 2-bottle choice			
Binge DID	C57BL/6J mice		Szumlinski et al., 2019
Operant self-administration	Sprague-Dawley rat		Nieto and Kosten, 2017
LORR	α6 KO mice	↑ sedation in KO mice, *n.d.* between sexes.	Kamens et al., 2012
CPP		↓ sensitivity to CPP, *n.d.* between sexes.	Guildford et al., 2016
Locomotor activity + varenicline pretreatment	DBA/2J mice	↓ alcohol-induced locomotion with varenicline pretreatment, *n.d.* between sexes.	Gubner et al., 2014
Binge DID + bPiDi pretreatment	C57BL/6J mice	↓ alcohol consumption with bPiDi treatment, *n.d.* between sexes.	Kamens et al., 2017
**Alcohol and nicotine co-consumption**			
3-bottle choice, chronic access	C57BL/6J mice	↑ alcohol and nicotine consumption in female vs. male. *n.d.* in nicotine consumption upon alcohol abstinence. Males ↓ alcohol consumption when presented alongside nicotine; *n.d.* in females.	O’Rourke et al., 2016
		↓ alcohol consumption when alcohol solution contains quinine, *n.d.* between sexes. Females ↑ nicotine consumption when consuming less alcohol.	DeBaker et al., 2020a
Operant alcohol self-administration + nicotine pretreatment; reinforce demand	Wistar rat	Baseline: ↑ reinforcement index in males vs. females. Nicotine pretreatment: ↑ perseverative responding only in females.	Barrett et al., 2020

*bPiDi, N,N-decane-1,10-diyl-bis-3-picolinium diiodide; CPP, conditioned place preference; DID, drinking-in-the-dark; KO, knockout; LORR, loss of righting reflex; *n.d.*, no difference.*

Nicotinic acetylcholine receptors have been implicated in alcohol-induced sedation, which is typically measured through the loss of righting reflex (LORR) assay. Mice lacking the α6 nAChR subunit are more sensitive to alcohol-induced sedation (Kamens et al., 2012) and less sensitive to alcohol conditioned place preference (Guildford et al., 2016), with no reported effect of sex. The α4β2^∗^ and α6^∗^ partial agonist varenicline can also increase LORR duration (Kamens et al., 2010) and block alcohol-induced locomotor activity (Gubner et al., 2014) similarly in male and female mice. The α6β2^∗^ nAChR antagonist bPiDi reduces binge alcohol consumption similarly in male and female mice but does have a sex-specific effect on locomotor activity, with bPiDi alone causing locomotor suppression in females and not males (Kamens et al., 2017).

One potential connection between alcohol behavior and the nAChR system is alcohol withdrawal. Residues in the α4 nAChR subunit gene that render the α4β2^∗^ nAChR subtype sensitive to alcohol potentiation are implicated in the severity of alcohol withdrawal (Butt et al., 2003, 2004). The α4β2 nAChR subunit is also potentiated by estradiol (Curtis et al., 2002), which could potentially contribute to some of the sex differences observed in alcohol withdrawal (Devaud and Chadda, 2001; Silva et al., 2009; Forquer et al., 2011). More studies are clearly needed to thoroughly characterize the influence of nAChRs on alcohol behaviors in female rodents.

## Sex Differences in Alcohol and Nicotine Co-Consumption

While the comorbidity of alcohol and nicotine use disorders has been widely studied in human patients, less research has been performed on the co-consumption of both substances in animal models. Our group has pioneered a 3-bottle choice paradigm in which male and female C57BL/6J mice will voluntarily consume both alcohol and nicotine, allowing for the study of compensatory consumption following removal of alcohol or nicotine from the cage (O’Rourke et al., 2016; DeBaker et al., 2020a, b). The 3-bottle choice procedure has also been adapted for use in female alcohol-preferring (P) rats to study the effect of pharmacological agents on co-consumption (Waeiss et al., 2019). More technically challenging paradigms include training rats for IV nicotine self-administration while simultaneously having access to alcohol solution in a 2-bottle choice paradigm (Maggio et al., 2018), and training rats for IV nicotine self-administration with simultaneous self-administration of alcohol (Lê et al., 2010). However, these last two rat studies were performed only in females and males, respectively.

While female C57BL/6J mice generally consumed more of both alcohol and nicotine in the 3-bottle choice model, both sexes increased their nicotine consumption following removal of the alcohol bottle, suggesting both male and female mice will compensate for alcohol abstinence by consuming more nicotine (O’Rourke et al., 2016). However, a sex difference was apparent when examining the total alcohol consumed in a 2 versus 3-bottle choice paradigm: male C57BL/6J mice drank less alcohol when it was presented alongside nicotine, whereas female C57BL/6J mice consumed the same amount of alcohol with or without concurrent nicotine access (O’Rourke et al., 2016). In a follow-up study, our group performed a 3-bottle choice experiment in which quinine, a bitter tastant, was added to the alcohol solution. While both male and female mice dose-dependently reduced their alcohol consumption in response to increasing concentrations of quinine, only female mice compensated for reduced alcohol consumption by increasing their nicotine intake (DeBaker et al., 2020a). Similarly, a recent report used a reinforcer demand analysis in which Wistar rats that were trained to self-administer alcohol were first pretreated with nicotine followed by an exponential reinforcer demand method to measure the reinforcement index of alcohol following nicotine treatment. In this study, male rats displayed higher alcohol reinforcement at baseline, whereas low-to-moderate doses of nicotine increased perseverative responding for alcohol infusions only in females (Barrett et al., 2020). These data suggest that female rodents may exhibit unique behavioral responses to co-consumption of alcohol and nicotine, which could have important consequences for treating comorbid alcohol and nicotine use disorders.

## Sex Hormones as Modulators of Nicotinic Acetylcholine Receptor Expression and Function

### Direct Receptor Interactions

While few studies to date have investigated the direct interaction between sex hormones and nAChRs, there is evidence from *in vitro* models that suggest estradiol (E2), progesterone, and testosterone can directly modulate different nAChR subtypes. Human α4β2 nAChRs expressed in oocytes exhibit robust potentiation following application of micromolar concentrations of estradiol (Curtis et al., 2002). A series of pharmacological experiments show that E2 enhances apparent acetylcholine affinity for the α4β2 subunit interface (Curtis et al., 2002), a feature that appears unique for E2 as both progesterone and testosterone have been shown to functionally inhibit human or rat α4β2 nAChRs *in vitro*, respectively (Valera et al., 1992; Paradiso et al., 2000). Further characterization of the interaction between E2 and α4β2 nAChRs localized this interaction to a discrete sequence in the extreme C-terminal domain of the α4 subunit, which can be introduced to other nAChR subtypes to achieve similar potentiation of acetylcholine currents (Jin and Steinbach, 2011).

The studies described above were performed in oocytes injected with human nAChR cDNA, and there is limited data available on the effect of E2 on nAChRs from rat or mouse. However, the gene encoding for the α4 subunit in rats is reported to undergo alternative splicing with 2 potential variants, each of which differs only in the 3 most terminal residues on the C-terminus that have been implicated in E2-mediated potentiation (Connolly et al., 1992). While studies reported that E2 inhibits rat α4β2 nAChR function when expressed in oocytes (Paradiso et al., 2000; Damaj, 2001), this may actually reflect the variant of the rat α4 subunit which does not contain the amino acid sequence at the C-terminus necessary for E2 potentiation (Curtis et al., 2002; Jin and Steinbach, 2011). To the best of our knowledge, no study has directly examined the expression pattern of α4 nAChR splice variants in the rat brain, or directly tested whether the 3-residue substitution present in the rat α4 variant actually confers sensitivity to E2 potentiation. There is also no data on direct E2 interactions with mouse nAChRs, or whether the gene encoding the α4 nAChR subunit in mice undergoes the same alternative splicing as in rats. Directly testing the effects of hormones on nAChR signaling *ex vivo* remains technically challenging due to the heterogeneity and diverse expression profile of nAChRs in mammalian tissue. Accordingly, further physiological studies on the interaction between different nAChR pentamers and E2 *in vitro* will be an important next step toward understanding the interplay between sex hormones and the nAChR system in both rodents and humans.

### Nicotinic Acetylcholine Receptor Expression

Sex hormones can have both short- and long-term effects at the cellular level, with E2 specifically being known for its ability to influence gene expression through the regulation of nuclear transcription factors such as CREB (Björnström and Sjöberg, 2005). As discussed previously, very little is known regarding the transcriptional regulation of nAChR subunits. However, there is correlational evidence to suggest that sex hormones may additionally play a role in regulating nAChR expression. Two classic studies found α7 nAChR binding in the suprachiasmatic nucleus was eliminated in female rats following gonadectomy and restored by E2 treatment (Miller et al., 1982, 1984). E2 administration has also been shown to increase α7 nAChR expression in serotonergic and noradrenergic nuclei in macaques (Centeno et al., 2006). In light of the recent study showing divergent nicotine consumption patterns in male versus female α7 knockout animals (Bagdas et al., 2019, Table 2), these data indicate that the α7 nAChR subunit may be an important contributor to sex differences observed in nAChR-dependent behaviors. In female human patients, availability of β2^∗^ nAChR binding sites in the cortex and cerebellum was negatively correlated with progesterone levels on imaging day, suggesting sex hormone signaling may also modulate the expression of high-affinity nAChR subtypes (Cosgrove et al., 2012).

Moreover, chronic nicotine treatment in female rats can reduce both circulating E2 and the expression of downstream signaling molecules including estrogen receptor β, CaMKII, and pCREB whereas these adaptations are reversed following E2 treatment (Raval et al., 2011, 2012). As several other studies have also found sex differences in nAChR expression both at baseline and following chronic nicotine treatment (summarized in Table 1), these data highlight the possibility of a direct relationship between E2 signaling and nAChR expression. Indeed, ovariectomized female rats show substantially higher nicotine-induced dopamine release from prepared striatal synaptosomes following E2 treatment, even compared to male gonadectomized rats given equivalent doses of E2 (Dluzen and Anderson, 1997). As nicotine-induced dopamine release in the striatum is mediated directly by nAChR signaling (Berry et al., 2015), this study further implicates E2 in the regulation of nAChR expression in the mesolimbic dopamine system. Additionally, the lack of effect in males treated with E2 further suggests a sex-specific bidirectional role for E2 in the rat brain, a report similar to those obtained in other laboratories (Boulware et al., 2005). Further delineation of the regulatory mechanisms underlying nAChR transcription both at baseline and following chronic nicotine treatment will be a necessary next step in understanding the influence of sex hormones on nAChR expression.

### Nicotine Behaviors

Importantly, a role for sex hormones in nAChR expression and function does not necessarily implicate behavioral variability across the estrous cycle for rodents. The misconception that outcome measures in female rodents are inherently more variable than males due to the estrous cycle has been generally disproven (Beery, 2018). Several studies have shown nicotine and alcohol consumption or self-administration does not vary by estrous stage (McKinzie et al., 1998; Donny et al., 2000; Priddy et al., 2017); however, another study showed an increase in nicotine seeking after nicotine abstinence in adolescent female rats during estrous compared with non-estrous (Lynch et al., 2019). There are other notable sex differences in nicotine pharmacology and behavior that appear to depend on sex hormone signaling. In Sprague-Dawley rats, repeated IV infusions of nicotine resulted in 10× higher plasma concentration of nicotine in females compared with males (Harrod et al., 2007). The difference was eliminated by gonadectomy of both sexes, suggesting that multiple sex hormones may influence nicotine pharmacokinetics following chronic nicotine infusions. Nicotine-induced antinociception was also attenuated by pretreatment with progesterone or estradiol in female ICR mice, with no effect of testosterone pretreatment in males (Damaj, 2001). An additional study utilizing spontaneous withdrawal also found that increased anxiety-like behaviors and increased corticotropin-releasing factor in female rodents was eliminated by ovariectomy, suggesting that circulating sex hormones may influence some of the physiological and behavioral aspects of nicotine withdrawal (Torres et al., 2014). This has been further tested using supplementation of estradiol and progesterone to ovariectomized female rats prior to nicotine withdrawal, with estradiol promoting and progesterone attenuating anxiety-like behavior (Flores et al., 2020). Although results from studies in women smokers who either have their hormone levels assessed on test day or are undergoing hormone therapy are mixed (Carpenter et al., 2006; Sofuoglu et al., 2009; Saladin et al., 2014; Allen et al., 2019), further study on the relationship between circulating hormone levels and nicotine behaviors will provide crucial information for helping women who smoke achieve and maintain abstinence.

## Concluding Remarks

Despite the historic exclusion of female animal subjects in preclinical neuroscience research, emerging evidence strongly supports an effect of biological sex in the nAChR system of both rodents and humans. In this review we have outlined preclinical data suggesting that sex plays a multifaceted role in nAChR regulation and nicotine-dependent effects, from nAChR subunit expression to complex behavioral phenotypes. At the molecular and cellular level, how sex influences nAChR subunit expression, at baseline and after chronic drug exposure, appears to differ depending on the nAChR subunit, which then can manifest as sex differences in nAChR subtype expression and receptor effects. At the behavioral level, sex influences numerous nicotine and alcohol phenotypes from consumption and self-administration to withdrawal-induced anxiety. In general, female rodents self-administer more nicotine and alcohol than males, and show greater nicotine-withdrawal induced responses compared with males; however, the effect of sex in many other nAChR-related behaviors have yet to be investigated. The majority of rodent studies that report sex differences did not track sex hormone status in male or female animals, suggesting that developmental sex differences, rather than circulating sex hormones, have an important role; however, there is evidence that sex hormones can influence nAChR expression and nicotine responses, and more study is needed to determine the exact roles of specific sex hormones. Possible directions for future studies are vast and include (1) direct interactions between sex hormones and different nAChR subtypes; (2) transcriptional and translational mechanisms underlying nAChR expression in both male and female rodents; (3) the effect of sex on nicotine and alcohol withdrawal; and (4) further characterization of alcohol and nicotine co-consumption in male and female rodents. The study of sex differences in nAChR-dependent phenomena is a rich and exciting area for future research. Further study of the way biological sex interacts with the nAChR system will be critical not only for the effective treatment of alcohol and nicotine use disorders, but also for a more complete and rigorous understanding of the fundamental principles underlying nAChR pharmacology.

## Author Contributions

JM wrote the manuscript. AL edited the manuscript. Both authors approved the submitted version.

## Conflict of Interest

The authors declare that the research was conducted in the absence of any commercial or financial relationships that could be construed as a potential conflict of interest.

## Publisher’s Note

All claims expressed in this article are solely those of the authors and do not necessarily represent those of their affiliated organizations, or those of the publisher, the editors and the reviewers. Any product that may be evaluated in this article, or claim that may be made by its manufacturer, is not guaranteed or endorsed by the publisher.
